# Patterns of computer and Internet use and its association with HIV knowledge in selected countries in sub-Saharan Africa

**DOI:** 10.1371/journal.pone.0199236

**Published:** 2018-06-27

**Authors:** Sanni Yaya, Bishwajit Ghose

**Affiliations:** School of International Development and Global Studies, Faculty of Social Sciences, University of Ottawa, Ottawa, Ontario, Canada; Universita degli Studi di Perugia, ITALY

## Abstract

**Background:**

Healthcare systems in Sub-Saharan Africa (SSA) are fraught with numerous governance and infrastructural issues including lack of access to quality care and health worker shortage. Policy makers are becoming increasingly interested in adopting novel technologies such as web-based interventions within the scope of e-Health to bridge the gaps in care delivery in a cost-effective and sustainable manner. Successful implementation of these policies is reliant on evidences regarding people’s access to these technologies, which are scarce for countries in SSA.

**Objectives:**

To 1) investigate the variation in the prevalence of accessing computer and internet across regional and socioeconomic groups, and 2) assess association between ever accessing computer and internet and knowledge of routes and risk factors of HIV transmission in selected SSA countries.

**Methods:**

We analyzed cross-sectional datasets from UNICEF Multiple Indicator Cluster Surveys. Participants were16,194 men and 39,121 women from Ghana, Guinea Bissau, Malawi and Zimbabwe. Main outcome variable was ever-accessing computer and Internet for any purpose. Associations were assessed by multivariable regression methods.

**Results:**

Lifetime computer usage in Ghana, Guinea Bissau, Malawi and Zimbabwe was respectively 21.5%, 13.4%, 12.3%, 28.4% among men, and 12.5%, 8.3%, 4.8%, 20.5% among women, and that of internet was 14.9%, 11.7%, 10.8%, 34% among men, and 6.4%, 6.9%, 4.2%, 21.6% among women in the aforementioned order. Participants who reported ever using computer and Internet were more likely to have higher knowledge regarding the transmission of HIV compared to those who did not.

**Conclusions:**

Prevalence of lifetime access to computer and Internet was considerably low in all four countries. Several socioeconomic factors appeared to be associated with the access to computer and Internet, addressing which might prove beneficial for the successful expansion e-Health in these countries.

## Introduction

Among the manifold challenges faced by the healthcare systems in SSA, poor public health infrastructure and human resource shortage are some of the most severe and persistent ones. As per the threshold for “critical shortage” of health workers set by World Health Organization (2.28 health workers per 1000 population), worldwide around 57 countries fall under this category, of which 36 are in SSA [1]. According to certain estimates, another 2.4 million skilled health workers are needed to meet the demand of health worker supply across the region [2] which accounts for about a quarter of the global disease burden with a meagre 3% of the global supply of health workers [3]. Lack of capacity to stay on par with the advancements in Information Communication Technology (ICT) leaves the regions subject to the so called ‘digital divide’, which can more appropriately be termed as ‘digital health divide’ in the domain of public health [4].

The World Health Organization defines eHealth as- the use of information and communication technologies (ICT) for health [5]. Application of eHealth, together with advances in health information technology marks the opening of new era in global public health, and is unfolding with unprecedented opportunities for global health promotion. A remarkable feature of eHealth is its capacity to enhance physician/provider-patient communication, patient engagement, enabling patients to better understand their health status which are the key components for promoting patient empowerment and more integrated approach to service delivery [6,7]. Although lagging behind other world regions, healthcare systems in SSA are increasingly being shaped by information and technological revolution and gathering momentum for eHealth and mHealth based health interventions [8]. Governments are showing increasing interest in investing eHealth and mHealth technologies with the view to building stronger health systems through the adoption of data-driven and digital solutions to the healthcare challenges meet the health needs especially of the underserved population. For instance, the Government of Ghana launched the national eHealth strategy in 2010, and as of 2014 about 22 eHealth projects were already at various stages of implementation [5]. In Malawi, electronic medical record system (EMR) is gaining increasing popularity for improving provider and management efficiency in the antiretroviral therapy clinics [9]. Internet is also appearing as a popular venue for acquiring and sharing medical information among patients, practitioners, and general population at large in an effective, time-saving and cost-effective manner. Thus, the emerging digital revolution is likely to have a profound impact on health expenditure and care delivery models as face-to-face interaction between patients and practitioners will become less common with the communication to be increasingly be mediated by electronic devices [10].

Although eHealth and mHealth technologies are widely embraced in SSA, little research attention has been paid to exploring the roots of the barriers to utilization of these facilities at population level. Several market studies are available providing the statistics on computer and Internet access, and national reports on the inequality mapping the digital divide, however evidence from the perspective of population health surveys are still scarce. Evidence regarding the enabling and limiting factors for utilization of basic facilities such as mobile phones and computer constitute a crucial component of the successful implementation and expansion of eHealth. This will help not only in informed decision making in the field, but also to form the basis for addressing the issues to promote the use of the technologies among the people. In the present study we aimed to measure the patterns of computer and internet usage among adult men and women in selected countries in SSA, and if the prevalence of usage varied across regional and socioeconomic factors. We additionally investigated if the users differed from non-users in terms of their knowledge regarding the risks/routes of transmission of HIV.

## Material and methods

### Data collection and survey characteristics

Data for the present study were obtained from the UNICEF Multiple Indicator Cluster Surveys conducted in Ghana (MICS round four, 2011), Guinea Bissau (2014), Malawi (2013–14) and Zimbabwe (2014). The program (Global MICS program) was developed by UNICEF in the 1990s to help countries generate internationally comparable data on a wide range of indicators on adult men (15–59 years), women (15–49 years), and children (0–5 years) for use in policies and programs and monitor progress towards the Millennium Development Goals (MDGs) [11]. The surveys were carried out by Ghana Statistical Service, Direcção Geral do Plano/Instituto Nacional de Estatística (INE) in Guinea Bissau, National Statistical Office in Malawi, Zimbabwe National Statistics Agency (ZIMSTAT) in Zimbabwe. Survey designs and sampling protocols were published by the representative institutions in each country [12–15].

### Variables

The main outcome variable in this study was ever-accessing computer and Internet. Participants were asked: “Have you ever used a computer?” and, “Have you ever used the Internet?”. The answers were categorized as Yes/No.

Additionally, we checked if having used computer and Internet was associated with higher knowledge level of HIV. This is possible to do as MICS surveys provide some basic indicators to assess the knowledge of HIV transmission among men and women. The following questions were analyzed to assess the level of knowledge about HIV transmission. 1. Can avoid AIDS virus by having one uninfected partner, 2. Can get AIDS virus through supernatural means, 3. Can avoid AIDS virus by using a condom correctly every time, 4. Can get AIDS virus from mosquito bites, 5. Can get AIDS virus by sharing food with a person who has AIDS, 6. Healthy-looking person may have AIDS virus, 7. AIDS virus from mother to child during pregnancy, 8. AIDS virus from mother to child during delivery, 9. AIDS virus from mother to child through breastfeeding. Scores were assigned as ‘1’ for each correct and ‘0’ for each incorrect answer. Total score ranged from 0 to 9. Mean score was calculated to check how what percentage of the participants, score above or below mean.

The explanatory variables of main interest were area of residence, wealth status and educational attainment. This was based on insight from previous studies for their use as proxy indicators of socioeconomic determinants of health. Few other covariates were also considered following the literature review and availability on the datasets. These were: Age (15–24, 24+), Area (Urban/Rural), Frequency of reading Newspaper (Almost every day/At least once a week/ less than Once a week/Not at all), Frequency of watching TV (Almost every day/At least once a week/ less than Once a week/Not at all), Frequency of listening to Radio (Almost every day/At least once a week/ less than Once a week/Not at all), Education attainment (None to Primary/Middle to Secondary/ Higher), Wealth quintile (Poorest/poorer/Middle/Richer/Richest) [4–7,9,16].

### Data analysis

Data analyses were carried out using SPSS version 22. Datasets were checked for missing values, outliers and were weighed prior to analysis. All analyses were stratified by gender. Prevalence rates of accessing to computer and Internet were presented as percentages. Following the descriptive analysis, cross-tabulation was performed with Chi-square tests to assess the bivariate association of computer and Internet usage with the socio-demographic and media use characteristics. The variables that were significant at p<0.25 were selected for multivariable regression analysis. All variables were entered into the regression model at the same time. Two separate models (for men and women) were run for each country. Secondly, we ran an additional regression model to assess the relationship between computer and Internet usage and HIV score. Thus, computer and Internet use were used as the dependent variables in the first regression analysis, and as explanatory variable in the second. Results of regression analyses were presented as odds ratios. Statistical significance was set at p<0.05 (two-sided). The Hosmer-Lemeshow and Nagelkerke R^2^ were used to test model fitness were, which indicated satisfactory fitness for all countries (0.39–0.68).

## Results

**Fig 1:**
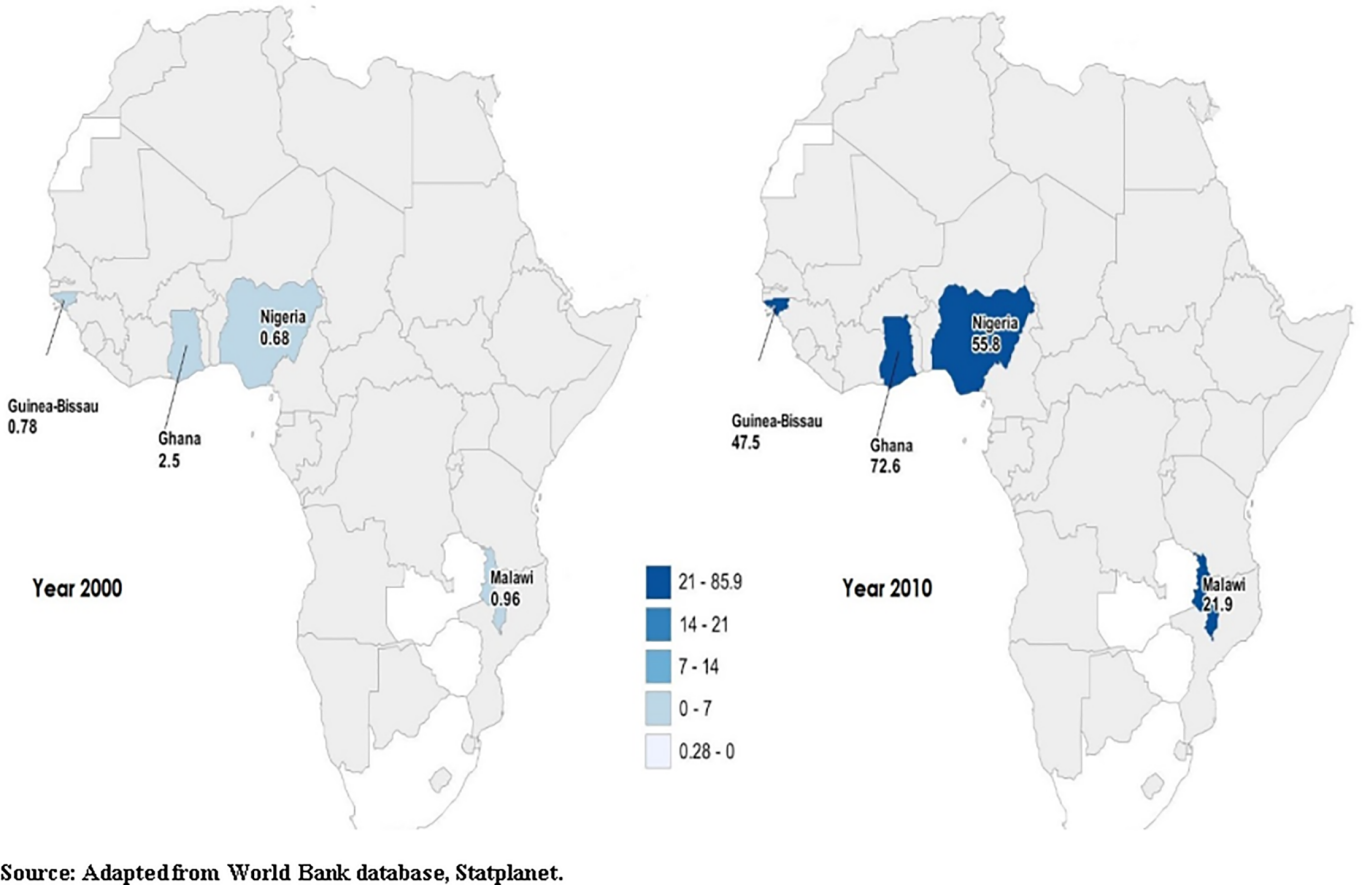
Trend in the prevalence of fixed and mobile phone subscription in the study countries.

Data on mobile phone were not available from MICS surveys. To provide general scenario of the mobile phone usage we used data from World Bank (Statplanet) resources for the years 2000 and 2010. Fig 1 below offers a comparative view of the density of fixed and mobile phones users for the years 2000 and 2010 indicating a considerable rise in the number of mobile phone users in all four countries.

### Descriptive statistics

In total 16,194 men and 39,121 women were included in this study. Basic demographic and socioeconomic characteristics of the sample population were presented in Table 1. In Guinea Bissau and Malawi, majority of the men and women were aged between 15 and 24 years, while in Ghana and Zimbabwe majority were aged above 24 years. The population was predominantly rural, comprising over three-fifth in most of the countries with four-fifth in Malawi (82.4% men and 83.8% women from the rural areas). Percentage of reading newspaper almost every day was highest for men in Zimbabwe (11.8%) and lowest for Ghanaian women (3.9%). Majority of the participants reported reading at more than once per week (except for men in Guinea Bissau). Regarding electronic media, more than a quarter watched TV almost every day (except in Malawi). In majority of the countries, the overall rate of listening to radio was higher than that of watching TV. Rate of reading newspaper and listening to radio were higher women men than women in all the countries. In all four countries, majority of the women had primary or below-primary level education, while men had higher rates of having middle/secondary and higher education. Regarding wealth status, percentage of men and women who reported living in poorest households were highest in Ghana (36.6% and 36.3% among men and women respectively) and lowest in Zimbabwe (15.3% and 15.8% among men and women respectively). Over two-fifth of the men and women in Guinea Bissau, and over a quarter in Malawi were living in poorest to poorer households.

**Table 1:** Basic demographic and socioeconomic characteristics of participants from selected countries in SSA. Multiple Indicator Cluster Survey, UNICEF.

	Ghana		Guinea Bissau		Malawi		Zimbabwe	
	Men (3,321)	Women (10,627)	Men (2,003)	Women (4,328)	Men (2,856)	Women (9,757)	Men (7,914)	Women (14,409)
**Age** (Mean. SD)	32.27, 12.86	30.24, 9.92	19. 4, 2.6	28.2, 9.3	28.17, 9.54	19.2, 2.7	29.4, 10.5	28.6, 9.2
15–24	34.9	33.2	57.2	52.6	58.9	53.6	37.9	39.1
24+	65.1	66.8	42.8	47.4	41.1	46.4	62.1	60.9
**Area**								
Urban	35.8	38.2	37.3	36.8	17.6	16.2	32.9	37.0
Rural	64.2	61.8	62.7	63.2	82.4	83.8	67.1	63.0
**Newspaper**								
Almost every day	4.8	3.9	14.6	6.7	11.7	3.8	11.8	4.9
At least once/week	9.6	13.1	36.2	20.1	13.4	12.4	20.9	14.2
>Once/week	11.0	17.4	22.3	27.5	23.2	25.2	27.7	23.7
Not at all	74.7	65.6	26.8	45.8	51.7	58.7	39.3	57.3
**TV**								
Almost every day	29.1	25.2	26.3	29.5	11.2	8.7	27.4	29.9
At least once/week	15.7	15.6	40.6	11.6	11.5	5.3	14.8	9.0
>Once/week	11.5	9.8	16.1	43.8	21.1	11.8	18.6	13.3
Not at all	43.6	49.4	17.0	15.2	56.2	74.2	39.2	47.8
**Radio**								
Almost every day	57.6	37.6	69.3	50.1	45.0	29.6	37.7	28.7
At least once a week	17.6	21.4	25.2	29.5	21.3	15.8	20.6	16.9
>once/week	10.6	13.7	3.6	17.8	17.3	19.3	16.7	17.4
Not at all	14.2	27.3	1.8	2.6	16.4	35.3	24.9	37.0
**Education**								
None-Primary	40.8	55.3	9.2	44.2	64.7	70.1	26.4	28.2
Middle/ Secondary	37.0	31.3	48.2	34.2	31.6	28.3	64.1	65.0
Higher	22.1	13.4	42.6	21.6	3.7	1.6	9.5	6.8
**Wealth**								
Poorest	36.6	36.3	23.8	25.0	16.0	19.3	15.3	15.8
poorer	19.6	18.9	22.0	21.6	18.4	19.3	17.1	16.8
Middle	14.5	15.4	23.1	20.7	19.4	19.8	19.5	17.5
Richer	15.8	15.2	17.4	17.7	22.0	19.3	24.9	23.1
Richest	13.5	14.1	13.7	15.1	24.2	22.3	23.2	26.7

### Cross-tabulation

Results of cross-tabulation calculating the percentages of participants ever using computer and Internet were presented in Tables 2 and 3 respectively. In Ghana, Guinea Bissau, Malawi, and Zimbabwe, the percentage of ever using computer was 21.5%, 13.4%, 12.3% and 28.4% among men, and 12.5%, 8.3%, 4.8% and 20.5% among women respectively. In all four countries, the likelihood of computer usage was higher among those aged above 24 years (except for women in Ghana and Zimbabwe), lived in urban areas, read newspaper at least once a week (except in Ghana), watched TV and listened to radio almost every day, had higher educational level and lived in wealthier households.

**Table 2:** Percentages of participants who reported ever using computer. Multiple Indicator Cluster Survey, UNICEF.

	Ghana		Guinea Bissau		Malawi		Zimbabwe	
	Men (21.5)	Women (12.5)	Men (13.4)	Women (8.3)	Men (12.3)	Women (4.8)	Men (28.4)	Women (20.5)
**Age**								
15–24	48.6	63.9	44.2	48.9	44.8	46.7	38.0	51.4
24+	51.4	36.1	55.8	51.1	55.2	53.3	62.0	48.6
p	< .001	< .001	< .001	0.077	0.077	0.001	0.455	< .001
**Area**								
Urban	68.3	74.5	79.2	87.7	53.2	59.4	67.0	70.6
Rural	31.7	25.5	20.8	12.3	46.8	40.6	33.0	29.4
p	< .001	< .001	< .001	< .001	< .001	< .001	< .001	< .001
**Newspaper**								
Almost every day	15.3	9.0	22.0	9.1	17.4	12.4	28.5	17.6
At least once/week	25.2	25.3	50.6	40.5	35.2	31.1	34.5	30.4
>Once/week	22.0	27.1	19.7	34.5	28.3	33.3	24.1	29.8
Not at all	37.5	38.6	7.7	16.0	19.1	23.2	12.9	22.2
p	< .001	< .001	< .001	< .001	< .001	< .001	< .001	< .001
**TV**								
Almost every day	57.6	59.8	52.8	43.0	42.2	47.1	54.5	59.0
At least once/week	21.1	18.6	34.9	38.0	14.3	10.8	16.5	11.1
>Once/week	11.1	9.4	8.6	16.5	19.8	14.4	10.9	10.6
Not at all	10.2	12.3	3.7	2.5	23.7	27.7	18.1	19.3
p	< .001	< .001	< .001	< .001	< .001	< .001	< .001	< .001
**Radio**								
Almost every day	72.0	53.7	84.0	65.4	61.0	58.1	41.4	32.2
At least once/week	15.5	22.6	14.5	26.8	18.8	13.3	21.1	19.1
>Once/week	6.0	11.7	1.1	7.5	14.6	15.6	14.2	17.5
Not at all	6.4	12.0	0.4	0.3	5.5	12.9	23.3	31.2
p	< .001	< .001	< .001	< .001	< .001	< .001	< .001	< .001
**Education**								
None-Primary	3.9	3.6	2.2	1.3	2.1	6.1	2.1	1.5
Middle/ Secondary	30.1	29.7	13.8	8.9	28.3	22.4	27.5	27.1
Higher	66.0	66.6	84.0	89.8	69.5	71.5	70.4	71.4
p	< .001	< .001	< .001	< .001	< .001	< .001	< .001	< .001
**Wealth**								
Poorest	7.3	5.8	5.6	2.2	2.6	1.3	1.8	2.9
poorer	11.1	8.8	5.6	4.5	1.9	3.6	5.7	4.9
Middle	15.8	15.3	9.7	6.1	7.8	6.1	10.3	8.5
Richer	25.5	22.0	27.5	19.0	15.9	12.3	27.1	21.7
Richest	40.3	48.1	51.7	68.2	71.8	76.7	55.1	61.9
p	< .001	< .001	< .001	< .001	< .001	< .001	< .001	< .001

N.B. p-values calculated from chi-square tests.

**Table 3:** Percentage of participants who reported ever using Internet. Multiple Indicator Cluster Survey, UNICEF.

	Ghana		Guinea Bissau		Malawi		Zimbabwe	
	Men (14.9)	Women (6.4)	Men (11.7)	Women (6.9)	Men (10.8)	Women (4.2)	Men (34)	Women (21.6)
**Age**								
15–24	45.8	56.4	42.1	48.5	38.9	42.4	38.3	44.0
24+	54.2	43.6	57.9	51.5	61.1	57.6	61.7	56.0
p	< .001	< .001	< .001	< .001	< .001	< .001	0.309	< .001
**Area**								
Urban	79.0	87.0	86.0	88.9	51.7	66.3	63.8	76.6
Rural	21.0	13.0	14.0	11.1	48.3	33.7	36.2	23.4
p	< .001	< .001	< .001	< .001	< .001	< .001	< .001	< .001
**Newspaper**								
Almost every day	19.0	13.1	26.1	10.7	14.8	14.4	26.2	17.9
At least once/week	28.2	31.6	52.7	40.5	35.1	30.9	33.7	32.0
>Once/week	21.4	30.7	16.4	32.3	30.4	34.8	24.5	29.9
Not at all	31.5	24.7	4.9	16.5	19.7	20.0	15.6	20.2
p	< .001	< .001	< .001	< .001	< .001	< .001	< .001	< .001
**TV**								
Almost every day	62.7	73.9	53.2	47.1	40.9	57.4	52.8	63.0
At least once/week	20.2	15.3	36.2	34.3	16.6	12.3	17.7	11.6
>Once/week	9.5	5.8	7.2	17.2	20.3	12.6	11.7	9.2
Not at all	7.7	5.0	3.4	1.3	22.3	17.7	17.9	16.2
p	< .001	< .001	< .001	< .001	< .001	< .001	< .001	< .001
**Radio**								
Almost every day	74.4	60.9	67.0	66.7	62.3	60.0	43.9	34.2
At least once/week	14.9	21.8	27.0	26.9	20.0	13.8	21.1	19.2
>Once/week	6.0	10.9	3.9	6.1	13.1	15.0	14.2	16.4
Not at all	4.6	6.4	2.1	0.3	4.6	11.1	20.8	30.2
p	< .001	< .001	< .001	< .001	< .001	< .001	< .001	< .001
**Education**								
None-Primary	2.2	1.7	1.3	2.9	2.2	5.3	4.3	2.7
Middle/ Secondary	20.8	15.1	12.3	14.8	27.0	25.7	23.8	26.5
Higher	77.0	83.2	86.4	82.3	70.8	69.0	71.9	70.9
p	< .001	< .001	< .001	< .001	< .001	< .001	< .001	< .001
**Wealth**				2.7				
Poorest	3.6	2.2	2.1	3.7	2.6	1.0	2.0	1.6
poorer	8.9	3.4	6.0	6.7	4.3	1.7	5.7	2.3
Middle	11.7	8.6	11.9	12.8	8.0	3.4	11.7	6.2
Richer	27.2	18.8	28.9	74.1	16.6	7.7	30.5	23.6
Richest	48.6	67.0	51.1	0.000	68.6	86.2	50.1	66.3
p	< .001	< .001	< .001	< .001	< .001	< .001	< .001	< .001

N.B. p-values calculated from chi-square tests.

Table 3 indicates the percentage of Internet usage was respectively 14.9%, 11.7%, 10.8% and 34% among men, in Ghana, Guinea Bissau, Malawi, and Zimbabwe, and 6.4%, 6.9%, 4.2% and 21.6% among women in the aforementioned order. Similar to computer usage, the likelihood of internet usage in the countries usage was higher among those aged above 24 years (except for women in Ghana), lived in urban areas, read newspaper at least once a week (except for men in Ghana and women in Malawi), watched TV and listened to radio almost every day, had higher educational level and lived in the richer to richest households.

### Multivariable analysis

Results of multivariable regression analyses (Table 4 and 5) showed that area of residence, education and wealth status were significantly associated with both computer and Internet usage. In all the countries, men and women who lived in urban areas had higher odds of ever using computer and Internet compared with their rural counterparts. As expected, the odds of using computer and Internet were remarkably high among participants with higher level of education, especially among women. In Ghana and Zimbabwe for instance, compared to women who had no to primary level education, those who had middle/secondary level education were 4.8 and 4 times more likely, and those who had higher education were 14 and 11 times more likely to have ever used computer. In Malawi, the odds of internet usage were about 9 times higher among and women with middle/secondary level education, and about 28 and 30 times higher among those who had higher than secondary level of education. Similar to educational status, household wealth status was also highly associated with computer and Internet usage with the odds being increasingly higher with each leap in quintiles.

**Table 4:** Odds ratios of computer usage across the regional and socioeconomic characteristics.

	Ghana		Guinea Bissau		Malawi		Zimbabwe	
	Men	Women	Men	Women	Men	Women	Men	Women
**Area** (Rural)								
Urban	1.131	1.240	1.244	1.180*	1.634	1.995	1.905	1.321
**Education** (None-Primary)								
Middle Secondary	5.884	4.765	3.027	4.38	4.729	4.105	5.115	4.188
Higher	9.910	13.551	5.996	7.104	8.461	6.081	9.836	11.256
**Wealth** (Poorest)								
Richest	8.110	4.767	13.705	9.329	5.283	7.309	13.212	6.346
Richer	4.954	3.233	11.738	5.657	10.412	3.511	5.653	5.035
Middle	3.531	2.245	9.168	5.206	3.633	2.750	3.921	3.389
Poorer	2.523	2.281	2.645	2.945	2.110	2.086	2.437	2.492

N.B.

* = Not significant. Reference categories are shown in parenthesis. Models adjusted for age and media use variables.

**Table 5:** Odds ratios of Internet usage across the regional and socioeconomic characteristics.

	Ghana		Guinea Bissau		Malawi		Zimbabwe	
	Men	Women	Men	Women	Men	Women	Men	Women
**Area** (Rural)								
Urban	2.073	1.859	2.438	1.508	1.845	2.170	1.915	1.539
**Education** (None-Primary)								
Middle/ Secondary	9.058	8.512	3.274	4.325	5.914	3.470	5.274	8.325
Higher	27.534	29.748	8.462	9.072	7.436	13.629	8.150	11.249
**Wealth** (Poorest)								
Richest	10.887	5.019	14.708	9.377	4.670	10.617	14.302	14.779
Richer	4.495	4.424	6.579	8.901	3.795	8.357	6.572	12.802
Middle	5.161	3.875	5.344	6.141	2.992	5.497	3.746	5.540
Poorer	2.322	3.038	1.957	5.516	1.797	3.711	1.996	2.654

Results also showed that those who ever accessed computer and Internet were significantly more likely to have higher knowledge regarding the routs and risks of HIV transmission (Table 6). In Malawi for instance, men and women who ever used computer were 83% and 72% more likely, and those who ever used Internet were 3.8 and 2.7 times more likely to secure higher than the mean score compared with those who never used.

**Table 6:** Odds of scoring equal or higher than mean score of HIV transmission knowledge among men and women according to computer and Internet usage status.

	Ghana			Guinea Bissau		Malawi		Zimbabwe	
	Men		Women	Men	Women	Men	Women	Men	Women
**Computer usage (**No)									
Yes	1.389		1.324	1.235*	2.225	1.825	1.724	1.548	1.290
**Internet usage (**No)									
Yes	2.094		1.035	3.092	2.609	3.789	2.714	1.998	0.989*

N.B.

* = Not significant

## Discussion

In the present study, we have analyzed country-representative survey datasets from UNICEF MICS to measure the prevalence of computer and Internet usage among adult population in Ghana, Guinea Bissau, Malawi and Zimbabwe. The results showed that the prevalence of lifetime usage of computer and Internet was low in all the countries, and differed across gender and countries. Men were more likely report lifelong using computer and Internet compared with women in all four countries. Highest rate of both computer and Internet usage were observed in Zimbabwe followed by Ghana, guinea and Malawi, a pattern similar to that of literacy status in these countries. The percentage of Internet usage in Malawi was low, especially among women. This low percentage was understandable given the poor literacy rates as about two-third of the men and more than two-third of the women had nil to primary level education, which was higher than in other three countries. Interestingly, the prevalence of Internet usage was higher than that of computer in Zimbabwe (28.4% vs 34% among men and 20.5% vs 21.6% and women).

In the multivariable analysis, several regional and socioeconomic factors such as area of residence, educational attainment and household wealth status appeared to be significantly associated with the usage of computer and Internet. Rate of lifelong accessing was lower among those who lived in the rural areas, and was lowest among those who had no or primary level educational. As expected, in all the countries that percentage of usage of both computer and Internet was highest among those who had highest educational qualification and those in the highest wealth quintile, which suggests the presence of a social divide (referred to the gap between the information rich and information poor within a society) in the access to these technologies. This comes to no surprise as even in the developed countries, the ownership and usage of digital technologies tend to be lower among the socioeconomically disadvantaged communities and in the remote areas with poor supply of electricity and connection to Internet [17].

As the findings further suggest, men and women who had ever used computer and Internet were significantly more likely to have higher knowledge regarding the risk factors of HIV transmission. Previous researchers suggest that socioeconomic disparities are linked with unequal access to and utilization of health information and technologies, which translates to lack of health awareness, inadequate knowledge regarding the risk factors, and adoption of risky behavior [18,19]. As in many other disciplines and spheres of social life, information technologies have brought about revolutionary transformations in the way health knowledge are being stored, shared and utilized across the global community. Growing body of literature affirms the role of electronic media e.g. health blogs and social networking sites in promoting healthy behavior through helping people making healthier choices without vising a healthcare facility [20,21]. The widespread use of the terms ‘Internet medicine’, ‘Dr Google’ provides a more concrete evidence of this role Internet is playing for consumer health information. Clearly, health information seeking/sharing remains one of the leading among all Internet based activities [22]. The segment of population lacking access to these resources thus remain deprived of the copious amount of health information being shared each day- free of cost.

Arguably, increasing access to technology alone cannot may not guarantee change in lifestyle behavior and health outcomes as the association is likely to be mediated by a wide variety of sociocultural influences [23,24]. Nonetheless, eHealth/mHealth can serve as efficient tools for acquiring and dissemination of health messages and enable the public to make informed choices about their health [25]. In SSA for instance, the control and intervention programs for highly infectious diseases (e.g. HIV/AIDS, malaria, tuberculosis), improving access to information about the risk factors, symptoms and routes of transmission is of critical importance for long-term success. Apart from the perspective of the patients, implementation of eHealth technologies will also require a trained workforce to embrace this changing paradigm in service delivery, and communicating with patients and other stakeholders across the continuum of care. Further research is therefore warranted to provide insights regarding the challenges and potentialities of the technology from both patent and provider perspectives.

Increasing computer ownership is a hard target to achieve for resource limited countries, especially among those in the bottom segments of the society. However, subscription of mobile and smart phones and Internet connectivity through network operators are constantly rising in SSA. As the countries are witnessing a rapidly expanding telecommunication market, it is facilitating the scope for providing health services online and through mobile apps and messages (SMS). While the financial and skilled labor challenges at the start-up phase of are significant, expansion of digital health market holds great potential to mitigate the human resource, infrastructure and facility crises. Embracing the benefits and full potential of e- and mHealth services will also require addressing the social inequalities- the so called digital divide. Therefore, sustainable implementation has to be facilitated by a concerted effort from social researchers, public health experts and the ICT sectors. Arguably, the ultimate success in digital health sector would rely chiefly to the extent policy makers can address the socioeconomic divide and capacity constraints, by converting the ideas and experiences into workable frameworks. Needless to say, a major overhaul in policy formulation and political will be necessary to meet the health agenda of such broad scopes, especially those of meeting the rising crisis of healthcare workers across the countries [26,27].

Our study has important strengths and limitations to mention. As far as we are concerned, this is first ever study to investigate the social divide in the context of accessing computer and Internet services among general public in selected countries in SSA. Population based studies in the areas of eHealth are limited in these countries. Absence of solid evidences prevents health decision makers to be informed of the factors that hinder the progress of health programs. It is worthy of mentioning that research capacity for conducting comprehensive surveys are limited for countries in SSA. Although international development partners such as USAID, UNICEF MICS are conducting large-scale studies across countries, many contextual and underlying issues unique to certain cultures remain unexplored. Locally tailored researches are hence necessary to gain a deeper knowledge of the challenges in the healthcare systems. One particular strength of this study is the large sample size, high quality of data, and uniformity of the surveys that has made the cross-country comparison possible. Data were country representative and hence finding are generalizable to the adult population.

Among the limitations are the self-reported and cross-sectional nature of the surveys which makes the findings subject to reporting bias, and prevent making any causal inference. As the data were secondary, we could not control the selection and measurement of the variables. Social divide was measured in terms of area of residence, educational and wealth status only, which may not capture the broader scope of the scope. There was no information on mobile phone use; therefore we extracted relevant data from World Bank database. Surveys were conducted several years back and hence may not reflect the current situation. Several other strong components such ethnic, professional, healthcare and social policies were not possible to account for. Moreover, there was no information on to what extent the usage of computer and Internet was for health information seeking purposes. Future studies should focus on exploring the pattern of using these services for health communication, and to what degree the information seeking translates to real life practices.

## Conclusion

The present study aimed to measure the prevalence of lifetime accessing to computer and Internet from an eHealth perspective in Ghana, Guinea Bissau, Malawi and Zimbabwe. Prevalence of ever access to computer and Internet varied by age, place of residence, education, wealth status as well as media use characteristics. Based on these observations, the findings point out the occurrence of a substantial social divide in the use of technologies, and calls for programmatic actions to address these issues so as to promote access to health communication technologies overall. This will lead to a better exploitation of the potentialities of eHealth, which will ultimately open avenues for more innovative solutions to the persistent healthcare issues the countries are confronted with. Further studies are recommended to focus on exploring people’s attitude towards this emerging healthcare paradigm.
